# Institutions of care, moral proximity and demoralisation: The case of the emergency department

**DOI:** 10.1057/sth.2015.10

**Published:** 2015-06-03

**Authors:** Alexandra Hillman

**Affiliations:** a School of Social Sciences, Cardiff University, 10 Museum Place, Cathays, Cardiff CF10 3BG, UK. E-mail: hillmanae1@cardiff.ac.uk

**Keywords:** demoralisation, emergency medicine, institutions of care, moral proximity

## Abstract

This article draws on concepts of morality and demoralisation to understand the problematic nature of relationships between staff and patients in public health services. The article uses data from a case study of a UK hospital Emergency Department to show how staff are tasked with the responsibility of treating and caring for patients, while at the same time their actions are shaped by the institutional concerns of accountability and resource management. The data extracts illustrate how such competing agendas create a tension for staff to manage and suggests that, as a consequence of this tension, staff participate in processes of ‘effacement' that limit the presence of patients and families as a moral demand. The analysis from the Emergency Department case study suggests that demoralisation is an increasingly important lens through which to understand health-care institutions, where contemporary organisational cultures challenge the ethical quality of human interaction.

## Introduction

This article develops Bauman's (1989, 1990, 1991, 1994) theories of morality and proximity and Fevre's (2000, 2003) theory of demoralisation to explore the increasingly problematic nature of relationships of care in public health services. There have been a number of recent cases in the United Kingdom in which vulnerable people have been failed by the institutions that exist to care for them: the most emotive and controversial being the scandal that emerged following the revelation of gross institutional negligence within Mid-Staffordshire National Health Service (NHS) foundation trust which resulted in many unnecessary deaths and a great deal of suffering for patients and families (see Francis Report, 2013). This scandal compounded reports highlighting the lack of basic care, dignity and respect for older people in UK hospitals (Care Quality Commission (CQC), 2011; Health Service Ombudsman, 2011).

The problem, that this article seeks to address, is what is the explanation for such a lack of care in the NHS? Popular propositions tend to focus on the morality of individuals: the most prominent example within the NHS is the contemporary pathologisation of the ‘uncaring nurse'. This article offers an alternative to the pathologisation of the individual. By drawing on data derived from a case study of a UK emergency department (ED), this article highlights the increasingly problematic nature of the interactions that occur between staff and service users. These interactions are situated in their organisational context, to show how they are partly produced in response to the institutional structures and systems of New Public Management (NPM).

Public institutions of care, like the NHS, embody multiple and often competing sets of values relating to their purpose in serving the community. For example, health services may abide by a commitment to patient choice, a commitment that may have the potential to reduce care quality (Mol, 2008); or, services may be built around an ethic of care that could inadvertently compromise an ethic of justice (Hoggett, 2006b). Such dilemmas and conflicts are further complicated by attempts, particularly from the previous New Labour government, to depoliticise public institutions, to ensure impartiality, fairness and a focus on ‘what works' (Clarke *et al*, 2000). These attempts privilege technical aspects of providing goods and services and can result in the commodification of relationships between care providers and service users, hollowing out their moral and ethical meaning (Hoggett, 2006a). Finally, there has been an intensification across the developed world of systems and procedures that seek to manage risk in public institutions by limiting individual discretion in decision making in favour of ritualised tasks, performance targets and protocols (Checkland *et al*, 2004; McDonald *et al*, 2006; Brown, 2008; Brown and Calnan, 2009; Heath, 2010). Academic commentators have grouped these multiple and complex developments under the label NPM. NPM essentially adopts private sector forms and practices and places them at the heart of state sector service delivery (Du Gay, 2000; Pollitt and Bouchaert, 2000; Dent *et al*, 2004; Cooke, 2006; Hoggett, 2006a, 2006b).

For the NHS, these developments have been particularly dramatic. In recent years the NHS has experienced fundamental change, not just in relation to the management of risk and rationalisation, but also because of increasing levels of privatisation (Pollock, 2005), most recently endorsed in the 2012 Health and Social Care Bill under the guise of ‘re-commodification' (see Scamber *et al*, 2014). The impact of such change on NHS practitioners has been to generate new, distinctively managerial responsibilities that, some commentators suggest, shift the responsibility for the distribution of increasingly scarce resources away from governments and onto clinicians and, in some cases, patients themselves (Maruthappu *et al*, 2010). The ED is a focal point for political and public concern over NHS provision. Its unique position as both a service open to the community *and* a gatekeeper to acute hospital beds means that gaps in service provision, and the limited capacities to meet demand throughout the NHS, have come to be represented by the huge increases in the numbers of people arriving at the doors to the ED. This year saw many departments reach breaking point, with some deciding to close their doors on the grounds of patient safety (Osborne, 2015). It is therefore particularly poignant that the setting of the case study presented in this article is an ED, where the effects of NHS organisational change are intensely felt, politically sensitive and publically scrutinised.

Alongside the academic commentary on NPM systems, the sociology of medicine has provided further evidence for how these ‘institutions of modernity' (Bauman, 1994; Heath, 2010) in the NHS have had dysfunctional effects on the organisation and delivery of health care (see, for example, Alaszewski, 2006; Waring, 2007; Hillman *et al*, 2013). What is so far missing in both sets of literatures (the commentary on NPM systems or medical sociology's challenge to the changing political and organisational landscape in the NHS) is a direct utilisation of theories of morality and demoralisation to better understand the increasingly distorted and dysfunctional nature of the relationships between service providers and service users. There are, however, key pieces of work that have highlighted the relationship between values, ethics and organisational cultures. Menzies-Lyth's (1988) classic study, for example, shows the unintended consequences of institutional procedures developed to defend against what she describes as the essential anxieties inherent in nursing tasks including: the splitting up of the nurse–patient relationship, the depersonalisation of caring work, the reduction of discretion in decision making and a redistribution of responsibility for caring tasks. Her findings remain relevant to nursing care today (see Tadd *et al*, 2012; Hillman *et al*, 2013). This article contributes to the ongoing study of the changing nature of caring relationships in health-care work, by focussing specifically on the ways staff respond to their working environment. In particular, this article demonstrates how this response impacts upon staff's ability to draw upon moral categories in their relationships with service users.

An important body of work (see Hoggett, 2006a, 2006b; Hoggett *et al*, 2006), that helps contextualise the changing nature of these relationships, has highlighted the complexity of competing frames and networks (for example, institutional and biographical) that inform how those employed in public services relate to their work and the people they serve. By utilising ideas of morality and demoralisation, this research explores the presence and/or absence of a moral component within these competing sets of values. In particular, it questions if, how and when particular value systems become privileged over others. For example, what are the conditions in which the values of the institution takes precedence over service users?

To tackle these questions, this article explores Bauman's (1989, 1990, 1991, 1993) concepts of morality, moral proximity and practices of effacement to interrogate the interactions between staff and patients and to situate them within broader cultures of care. Bauman (1990) describes morality as the automatic responsibility for another person that occurs as a result of their proximity. Proximity impacts upon moral responsiveness (Walker, 1998) and is therefore constituted through relationships; these need not necessarily rely upon physical or emotional proximity but require some knowledge of the other person that will elicit a response. Bauman (1990, 1991) describes forms of social organisation that, even with the occurrence of face to face contact, limit the proximity of the person in ways that can restrict moral responsibility.

For Bauman (1990, 1991), it is not social organisation that is needed to tame the natural moral inadequacies of human beings; it is modern society that creates the means through which to limit proximity, creating a world where action is possible without being underlined by the human capacity of moral regulation. Fevre's (2003) demoralisation theory builds upon Bauman's central claim, that developments in modern society provide the conditions for a loss of morality in the shaping of our everyday lives. Fevre suggests that morality describes actions that arise from sensemaking based on categories of belief rather than knowledge. Without these resources we are only able to rely on guidelines from science or, increasingly, economic rationality. Fevre (2000, 2003) therefore provides a variation of Bauman's argument by suggesting that modern society creates an imbalance of sense-making resources available to people, setting the foundations for category mistakes in which people draw on the wrong resources to inform their actions. Demoralisation therefore has two meanings: first, a stripping away of morality or a reduction in moral actions and, second, a lost sense of purpose; it is both about morals and morale (Fevre, 2000). The increasing dominance of rationality as an essential grounding for the delivery of health care (Ahmed and Harrison, 2000; Clarke *et al*, 2000) means that health services have come to be governed by rational precepts of business economics that create multiple forms of sensemaking that exist in parallel to the resources of morality.

Following a brief account of the study, the remainder of this article is organised into four parts. The first part illustrates how processes of assessment in the ED create competing duties for staff. The second and third parts of the article – ‘creating distance, limiting moral proximity' and ‘re-establishing proximity' – build upon this ethnographic context and set out theoretical ideas that develop Bauman's concepts of moral proximity and effacement and show their relevance for understanding contemporary institutions of care. In the final part, the ‘Discussion' – the article draws together the previous analysis to suggest a (re)conceptualisation of moral proximity as a social accomplishment and finally, the question posed at the start of this article (How might we explain the growing problem of a lack of care in institutions such as the NHS?) is reconsidered in light of these theoretical developments.

## The Study

This ethnographic study of emergency medicine was carried out in a large inner city UK teaching hospital with a particular focus on the assessment, care and treatment of older people. The project ran over four years between 2004 and 2008 and received ethical approval from an NHS National Research Ethics Service (NRES) committee and research governance approval from the NHS trust taking part. Participants of the study included medical staff of all levels from health-care assistants to the clinical director, patients, patients' relatives or carers and managerial staff. All participants were given a pseudonym at the point of their first entry into fieldnotes. A separate document, accessible only to the researcher, kept a record of all participant names and their attributed pseudonym. The study comprised of 250 hours of observations in the ED and 35 qualitative interviews. The examples presented in this article are taken from fieldnotes of observations.

Observations were carried out across each distinct area of patient care within the ED and visits were arranged to cover the seasons, the days of the week and times of the day and night. The most intense periods of observation were carried out during the Winter months of 2006 and 2007 (November, December and January), and the Summer of 2007 (May, June and July). During these periods, visits to the ED were once or twice weekly. Timings of observations mirrored the shift patterns of staff, both doctors and nurses. The observations were flexible and unstructured but loosely took on two approaches: patients were tracked from their initial assessment to eventual admission or discharge; members of staff were shadowed while working their shifts. The meanings of the actions and interactions observed were further elicited and explored through both formal and informal interviews with staff and patients.

The 250 hours of observation accumulated over the course of the study included the tracking of 50 older patients (over 65) through their assessment in the ED. Although the focus of the study was on the experiences of older people, the assessment and treatment of patients under 65 became a central part of understanding the culture of the ED and older people's place within it. The observations focussed on the means with which staff accomplished categorising patients for priority of treatment, the negotiations that occurred during these clinical encounters and the meanings of these interactions for staff and patients (and their relatives). Observations also focussed on the organisational cultures of emergency medicine such as processes of clinical governance and professional practice, the socio-spatial organisation of the department and the people and materials within it, and formal and informal staff hierarchies and networks.

Both fieldnotes and interview transcripts were analysed thematically and this was undertaken simultaneously while carrying out fieldwork. This meant that emerging issues, such as the categorising of patients for priority of treatment, could be read and interpreted alongside broader institutional concerns of throughput and the rationalisation of resources. The researcher's position as observer was reflected upon to identify influences on the encounters they observed. For example, decisions over when to observe, how to stay attuned to the wishes of those being observed and when to withdraw altogether were continually negotiated in the field between the researcher, the patient and the staff participant. The ED is a fast paced environment, with strict rules and limitations on who can be where, when. It was therefore paramount that the researcher found a role, in each area of the department, which afforded them a degree of legitimacy, while enabling them to remain an observer. This often involved being enroled as a ‘student' or ‘assistant' by the staff themselves. These ethical and practical negotiations in the field were recorded in the fieldnotes to show how they informed the interpretation of meaning in the data.

The data collected together within themes were checked for the consistency and validity of interpretation. The constant comparative method (Silverman, 1993) was used to check the relationship between concepts and to build common themes. Initial analysis was discussed with practitioners and patients informally during fieldwork visits as a means of ‘respondent validation' (Bloor, 1978).

### The juxtaposition of duty

The focus for the analysis presented in this article is on the interactions that occur at the point of access to emergency medicine. The initial negotiations over accessing the ED occur during triage, a system set up to prioritise patients according to clinic need. Triage is the process in which the contestation and negotiation over accessing the resources of emergency medicine is most intensely governed (Gibson, 1978). Triage is a perfect description of Fevre's (2000) ‘mixed field': it organises patients according to categories of priority; priority is determined according to a complex interaction between clinical judgement, professional interests and the perception of patients' moral worth (for examples of patient categorisation, see Roth, 1972; Hughes, 1976; Latimer, 1999; Hillman, 2014).

Owing to its physical and symbolic location between publics in need and acute in-patient care, this section of the ED is particularly mediated by institutional concerns over increasing demand and efficient patient throughput. For example, ‘the patients' as a group to be assessed, treated and discharged have a significant presence for those working at the point of access to the ED. As a result, there is a more explicit responsibility for staff working in triage to organise and account for ‘the patients' as well as assessing and treating individuals.

All patients are automatically logged on to an interconnecting computer system called ‘Jonah'. Jonah provides a checking system for every ED patient at all stages of the assessment process. This information can be called upon to check the location or status of a patient, monitor staff performance or to ascertain the working practices of the unit as a whole, as one of the nurses explained to me:

The system aims to ensure that everyone is made responsible for working efficiently ‘cause with this, everyone is accountable ‘cause it knows at all times who's responsible for each patient in the department. (Minor Injuries, Winter 2006)

Such practices engender social adjustment in individuals according to the guidelines set out by the institution; adjustments and actions that can then be ‘re-described as evidence of their accountability' (Strathern, 2000, p. 4). These adjustments in behaviour shift the focus of staff's attention so that they attend to the tool itself, rather than the patients it supports (Coughlan, 2006).

The production of the initial assessment form, that is added to and developed by staff to form the patient record, contributes to the way patients are ‘inscribed' (Latour, 1986). When doctors ‘collect patients', they actually collect the two-dimensional material inscriptions of patients produced through the patient record, not the ‘three-dimensional subject' (Mort *et al*, 2003, p. 273). Materials and systems through which patients are inscribed also exist among standards, protocols and guidelines that shape how staff interact with patients during processes of assessment:

In a quiet moment I notice a red file on one of the desks called ‘National Triage Presentational Flow Chart'. This file seeks to provide symptom signs that will allow for a more accurate placement of patients into appropriate triage categories so that, as stated on the inside cover of the file, ‘the more severe pathologies are appropriately triaged'. Inside the file are plastic wallets containing individual flow charts for specific presenting problems that a patient may attend A&E with. These flow charts ask a series of questions and provide possible responses. By following the responses a patient may give through this flow chart, a triage category is reached. (Triage, Winter 2006)

These tools represent what Ahmad and Harrison (2000) call scientific-bureaucracy in which clinicians rely upon protocols and guidelines to rationalise processes of assessment according to external evidence. Tools for supporting clinical decision making create a further means through which staff perceive patients differently. The application of this technology effectively reduces the ‘mixed field' to one form of sensemaking (Fevre, 2000) that reduces the possibility for staff to build upon their embodied, tacit knowledge and those more qualitative skills of interpersonal communication (Nettleton *et al*, 2008) in which patients' personhood remains. That is not to suggest that the techniques of clinical governance create a workforce acting only according to the values of scientific-bureaucracy (which is itself only one of many competing, and often contradictory, set of institutional values). Staff interpret and attach meaning to governance processes in a multitude of ways that may be contrary to their institutional intentions (Brown, 2011).

The purpose of providing this description is to show how relationships of care are mediated by technologies of audit that embed institutional concerns of rationalisation and efficiency into staff's daily decision making. Contradictions arise at these sites of negotiation, where the problem of caring for ill people is juxtaposed with the responsibility to account for one's actions according to such institutional concerns. The organisation of ED work, therefore, creates the potential for losses in moral proximity between staff and their patients, so that staff become demoralised and patients' de-humanised. The next section illustrates the mechanisms through which staff cope with competing duties and shows how these coping mechanisms both respond to and sustain the ED as a demoralised social space.

### Creating distance, limiting moral proximity

Staff working at the point of access to the ED are burdened with responsibilities. It is the responsibility of staff, particularly nursing staff in this area, to not only treat and care for patients but to manage them. They must manage ‘the patients' both for physicians, who expect to collect patients pre-assessed with a category of priority assigned, and for the institution, that requires patients to be ordered and tracked through the ED system on the basis of efficiency targets and practices of accountability. Allen (1997) has suggested that nurses can usefully be thought of as boundary workers: their work is located among patients, with other professionals and providers and their competing understandings of illnesses and epistemologies of treatments, needing to be interpreted and co-ordinated. In the case of the ED, this boundary work can be extended to incorporate the institutional logics of accountability and efficiency.

In order to cope with competing duties, staff participate in practices of effacement (Bauman, 1991) that can render patients and families ‘faceless', thus limiting their presence as a moral demand. Practices of effacement are a consequence of staff's potential moral distress from what Peter and Liaschenko (2004) describe as the perils of proximity. They argue that proximity to patients compels staff to experience their moral responsibilities so that constraints to that responsibility become morally distressing. The responsibilities of staff to manage patients according to institutional concerns can mean that acting on the basis of a moral response is challenged. Processes of effacement therefore create a necessary distance and detachment for staff, reducing the potential for moral distress or anxiety (Menzies-Lyth, 1988).

The extract below is taken from a conversation between two triage nurses:

On this occasion there was a nurse I had not met before in charge of minor injuries. Nurse Harbury. I introduce myself to her and again described briefly my research interests. She was not unfriendly, but quite disinterested. After getting despondent with patients congregating in complaint at the door to assessment room 1, where she was based, she growls at a fellow member of staff, an older nurse called Sister Smith.

They have a chat about *‘what this job does to you after a while',*

She claims, *‘doing this job will drive you mad, you end up hating the patients'* She looks at me, the conversations seems very much for my benefit. Sister Smith responds by saying *‘you're too young to be feeling like that…you should be smiling sweetly at the patients still'.* (Minor Injuries, Summer 2006)

Owing to both the physical presence of patients congregating in the waiting area, and the specific role of ED staff working in triage to assess, assign categories and process patients, ‘the patients', as a faceless entity to be managed has greater significance. As Bauman (1990) notes, as soon as the other is cognized, they become an object causing a fundamental break in proximity. As a consequence of this effacement, patients can become reduced to a collection of parts or attributes that can be labelled, ordered and quantified.

Nurse Harbury explains that ‘Doing this job will drive you mad'. This statement occurs following a number of incidents that occur at the door to assessment room one, a place in which the experience of working within a continually contested domain (Hoggett *et al*, 2006) is intensely felt. It is a place where ethical dilemmas and conflicts arise and where the demands upon staff to manage resources and adhere to processes of accountability are high. This is because of staff's unique role in managing the flow of attending ED patients and their admittance onto acute hospital wards. The multiple available categories for sensemaking (Fevre, 2000) in these incidences are therefore squeezed out, so that staff are compelled to act according to organisational priorities that, at least momentarily, become privileged. The madness Nurse Harbury refers to is therefore the feelings that derive from the detachment and the distortions that occur in staff's relationships with their patients. What is it that is ‘hateful' about the patients? In this context, the nature of the relationship between patients and staff is combative, patients become an enemy. It is not individual patients that the staff ‘hate' but the patients as a group. The reducing of patients to a homogenised group in opposition – the patients – is further exacerbated by the concerns staff have over patient complaints and litigation:

When I arrived in the triage room, a couple of the nurses are talking. They acknowledge me and go back to their conversation. They are discussing a colleague who would seem to have been implicated in an investigation of a patient complaint: Nurse John: The problem is that it should never have happened that Sue (a junior nurse) was assessing the patient on her own. It's her word against his now.

Nurse Jane: (looking over at me) people would think it's us against them the way we talk. (Triage, Winter 2007)

This example was one of many in which staff discussed the need to protect themselves against ‘the patients'. The relationship between risk, trust and the culture of blame, and its ramifications in health-care settings, has received much sociological attention (Brown, 2008; Jones, 2009; Locke, 2009; Petrakaki *et al*, 2014 to name a few). Such a culture shifts value away from patients as persons to adhere instead to institutional logics that protect staff's position within the institutional culture. As a result, both patients and staff are rendered less than full moral subjects. The imbalance and reduction of sense-making resources (Fevre, 2000) available to staff to make decisions in their daily work hollows out the moral value that can be attached to it and subsequently distorts their relationships with patients; they become demoralised.

According to Bauman (1989), for people to be rendered less than full moral subjects, proximity must be replaced with social distance that can only occur through a physical or spiritual separation of the other. In the case of the ED, it is the permeation of instrumental rationality in the form of systems of accountability, resource management and institutional risk (Power, 1997) of litigation, into the actions and decision making of frontline staff that works to neutralise the ethical dimension of clinical work and allows for the spiritual separation that Bauman describes. The extract below illustrates the way one member of staff maintains such a separation:

An elderly woman, Mrs Preston who is in her 80s enters nursing assessment room one. She has a bloody nose and mouth and is holding a handkerchief to her face to try and stop the bleeding. She is slightly dishevelled and seems a little shaky. She is helped in by another woman who looks slightly younger than Mrs Preston. Nurse Harbury motions for her to sit down on the chair in front of her as she says hello. As Mrs Preston is sitting down she explains to the nurse that she fell down in the park.

Nurse Harbury*: Do you remember everything?* Mrs Preston: *What do you mean everything? Uh, yes I think so.* Nurse Harbury*: What happened after you fell, do you remember?* Mrs Preston: *I remember being in a neighbour*'*s house…* Nurse Harbury: (Interrupts) *So you remember being on the floor?* Mrs Preston: (Tentatively) *Yes*

After Nurse Harbury had finished examining Mrs Preston she asks her if she would like to clean up a bit at the sink, as she has quite a lot of blood over her face. Her friend looks surprised but helps Mrs Preston to the sink and they struggle together to attend to her bleeding face and mouth. When they are finished, Nurse Harbury looks up from her notes and hands her a dressing to hold on her face until she can get her stitches done. (Minor Injuries, Summer 2007)

Significantly, Nurse Harbury does not attend to Mrs Preston's face. This is particularly poignant as a person's face is symbolic of their personhood and therefore the intimate care and attention involved in the process of cleaning facial wounds may break the possibility of moral distancing.

The shifting of patienthood on the basis of inscriptions that constitute patients in particular ways (Mort *et al*, 2003) is a significant method through which patients can be transformed into an other. The permeation of rationalisation into the work undertaken by staff working at the point of access to the ED, in the case below, constitutes the demotion of the patient from a full moral subject to a ‘faceless' entity characterised by specific attributes:

Following some difficulty with a mentally ill patient who had refused to leave the A&E department, there was a young woman with a cut on her ankle waiting to be seen by a doctor who the nurses believed to be a self-harmer. She has been waiting a considerable amount of time and had repeatedly knocked on the door to the assessment room, which added to the annoyance of the staff who had been ignoring her knocking. After the fourth or fifth time, Sister Smith opens the door and said *Look I'm with a patient at the moment. I will open the door when I'm ready to and not before.*

The young woman was clearly frustrated and responded by saying that she had been told to knock on the door by the reception staff. Sister Smith does not call the patient when she has finished dealing with the current patient. An hour later the woman left. (Triage, Winter 2006)

The nurses' suspicions that this woman had cut herself meant that she was left to wait considerably longer than other patients with a similar injuries. This incident responds in part to the competing sets of values and dilemmas that staff must negotiate in their relationships with service users. The value of compassion and care towards this patient in need is complicated by the responsibility towards all patients needing treatment and care. There are however complicating value systems at play in this example that reflect both professional and institutional interests. As has been shown in previous work (Jeffrey, 1979; Dingwall and Murray, 1983), those patients attending the ED with problems they are deemed to be responsible for are perceived negatively by staff and often experience forms of punishment including longer waiting times, fewer tests or examinations and sometimes even refusal of treatment. The problem this girl attends with is transformed into an attribute that effaces her. Not only is she part of the patients as a group in opposition, but she is a ‘bad' patient. These processes of effacement are sustained by the culture of rationalisation in the ED towards the treatment of acute trauma patients. Such a culture shapes staff's perceptions of patients so that specific traits attributed to them (Armstrong, 1983) can become tools enabling staff to distance themselves from patients' personhood.

To act upon such specific traits allow the staff to avoid moments that may induce morally significant effects. The attribution of moral categories is rarely given as a response to patients' personhood; rather these categories provide the means through which patients can be reduced to types. The following two extracts illustrate this phenomenon:

After lunch there were a few junior doctors gathered in Assessment room one, talking about what shifts they were on. They talked about how tired they were and how they weren't able to do anything other than sleep and work. One of the male doctors, Doctor Glass turned to me and asked, ‘so who are you, are you a student? I replied by saying yes, but not a medical student. I told him about my research, in the same way I had described it to Nurse Morris, that I was interested in Older patients who attend A&E, as although they are in need of emergency medical care, their problems are often more complex and may relate to chronic conditions and their social circumstances as well as their emergency medical needs. Dr Glass responded with: *So you're interested in the social. You'll wanna go to the trolley bay. That's where the social go. They're what the cynical, depressed medical students call crap* (he looked at the others and laughed and they smiled and laughed with him).

Whilst describing my research to another doctor in the assessment room, Nurse Price who had become a useful source of information when on duty, told me that what I am really interested in are the ‘a-copias'. I look confused and he goes on to explain that in medical terminology every word that begins with an ‘a', the ‘a' refers to without/nothing and so an ‘a-copia' is someone who can't cope. He chuckles and says *it's probably made up but it sounds good doesn't it?* (Triage, Winter 2006)

These extracts illustrate how perceptions of patients' identity or personhood are re-constituted as moral categories or traits that define patients as set types. These moral categories are constituted according to professional and institutional interests. Professional interests favour cases that are novel and provide possibilities for clinical intervention that necessitates skill and technology (Becker *et al*, 1961; Jeffrey, 1979). ‘Acopias' are those deemed unable to cope, who are deemed to have little or no demonstrable clinical problem. ‘Socials' are deemed to have problems that are a direct result of their social circumstances, (this can include their age) which negates their clinical needs. And the ‘crap' refers to those cases deemed to be minor or mundane. These patients are attributed such negative labels because they do not provide ‘good clinical materials' (Latimer, 2000) for staff to demonstrate clinical competency. The value (or lack of value) attributed to these patients is therefore, in part, a response to professional interests.

Along with professional interests, relationships between staff and patients are also informed by the institutional logics of emergency medicine. These logics necessitate that patients have a quick and measurable response to clinical intervention (so that they fit better within performance targets and efficiency tools) and fit the increasingly rationalised definition of the ED patient: the sufferer of an acute trauma (Hillman, 2014), thus re-establishing a definition of emergency medicine as a distinct speciality, ensuring greater status, power and potential for resources. For staff, these labels not only respond to the culture of medical work and the institutional concerns of emergency service provision, they also provide a means with which to efface those patients who may otherwise remain present as a moral demand. The proximity of the ill older person waiting on a trolley for an acute bed, for example, is instead reduced to another ‘social' who ‘shouldn't be here', lessening the potential for such patients to induce moral distress.

### Re-establishing proximity

The previous examples have shown how staff engage in practices that distance themselves from their patients as full moral subjects. However, there are moments when this distancing is challenged and proximity restored. The following example describes a situation in which Sister Brown's practices of effacement are challenged:

The first patient I observed was a sixty six year old woman, Mrs Jackson, who came in for a twisted ankle and foot. She explained that she had done it while getting out of her son's car at the cinema the night before. There are no obvious signs of swelling or bruising but the woman appears to be in a lot of pain. Mrs Jackson: *Honestly, I've been crawling around the house on my bum… I can't put any weight on it at all* Sister Brown: *Well, just to warn you. If your foot isn't broken you'll have to put weight on it and walk on it properly otherwise it won't heal.* Later, following an ex-ray of the patient's foot …. Sister Brown: *It's not broken so you'll need to take regular pain relief. The best is to take a combination of paracetomol and anti-inflammatory which you can take together three times a day. For the first couple of days elevate it, put an ice pack on it but make sure its wrapped in something don't put it straight on the skin and make sure It's for no longer than 10minutes in any hour. After a couple of days start trying to walk around on it.* Mrs Jackson: *What about driving?* Sister: *I wouldn't drive because with the pain you're having you won't have full control of the car* Mrs Jackson: (beginning to look upset) *My husband's in a care home you see and I drive to visit him a couple of times a day.* Sister: *What about your son who brought you in today, does he drive, could he not take you?* Mrs Jackson: (getting more upset) *He's going back to London later today, he was just visiting* Sister: (A little more sympathetic) *Ah, oh dear. It makes life difficult doesn't it?* Mrs Jackson: (Begins to cry) *How will I get to see him?* Sister Brown comforts Mrs Jackson by putting her arm around her, Mrs Jackson immediately seems better in response to this gesture. Mrs Jackson: *I'll just have to get taxis I suppose*

Sister: *Well after a couple of days the pain should have eased a lot, you could try driving then.* (Minor Injuries, Summer 2007)

In this example Sister Brown initially remains distant to Mrs Jackson, seeming quite dismissive of her explanations of pain and ‘crawling around on her bum'. However, through Mrs Jackson's sadness about the difficulties she will have in seeing her husband, Sister Brown begins to soften. The introduction of emotion into the interaction between Sister and patient, along with the patient's own accounting for her circumstances and her resolve to make the best of things – ‘I'll just have to get taxis I suppose' – begins to chip away at Sister Brown's earlier attempts to keep Mrs Jackson at a distance and shifts the conditions of their relationship. Malone (2003) suggests three types of proximity in relation to nursing care: physical, narrative and moral. In order for moral proximity to endure, a physical nearness to the patient's body and understanding of the patient's narrative are necessary so that the nurse may engage with the patient in their particularity. In this case, Mrs Jackson's story distinguishes her from ‘the patients' as a group, helping her regain her particularity and thus her personhood (Bauman, 1989).

The following example describes the last moments of a crisis in which a man collapsed in the waiting area of the ED:

When eventually the nurses began to take him towards resus' (the resuscitation room) the man managed to speak and asked if he was safe to which Nurse Claire replied *yes, we're going to look after you don't worry.* He then asked *am I going to die* to which she replied *No you're not going to die, not while I'm here.* Nurse Stuart then said *‘you're definitely not going to die here.* Nurse Claire: *Far too much paper work for us.* (Corridor between the waiting area and resuscitation, Winter 2006)

Although this extract may seem cold and insensitive, the context was rather different. The informality that such a joke engenders immediately alters the context of the situation so that the man's panic subsides and he is reassured. Moral proximity and moral response is enabled through the nurses' re-configuration of the rational systems that have worked previously to distance them from patients. The need to make an automatic response is essential to maintain proximity and re-establish the response to the patient pre-cognition (to use Bauman's term) or in parallel to cognition (to use Fevre's idea of the mixed field).

The important question to ask about this incident is: how is Nurse Claire able to re-frame the institutional concerns of record keeping and accountability (too much paper work for us) to reassure this man? Perhaps the immediacy of this patient's need means that the inherent anxieties of the nursing task (Menzies-Lyth, 1988) cannot be defended against through an adherence to organisational processes and protocols. Instead, Nurse Claire responds directly to the proximity of nursing work.

This subversion of the institutional priorities that shape staff's experiences of assessing and treating their patients is further exemplified in the extract below:

The National Triage Presentational Flow Chart file was used during an assessment of James, a young man who had attended A&E due to his hypo-glycaemia. Nurse Peters picked up the file and turned to the patient and said *let's try and get you through a bit quicker*. After looking at the presentation flow chart on diabetes, Nurse Peters filled in the triage assessment form. After the patient left the assessment room Nurse Peters looked at me and commented that *I did him a favour* … *tried to get him seen a bit quicker.* (Triage, Winter 2006)

In this example, Nurse Peters decides that this patient needs and/or deserves (values attributed to both clinical need and moral worth are likely to be at play) to be ‘seen a bit quicker'. Unfortunately, the background to this case, the taking of the patient's history and the interactions between the nurse and the patient, were not observed, so the full context of the encounter is unavailable. Perhaps the patient was able to present his case in a way that ensured him greater priority (Hillman, 2014), or perhaps Nurse Peter's own personal history meant that he attached particular value to the needs and/or circumstances of this young man's attendance (Hoggett *et al*, 2006). There are also many, diverse aspects of organisational culture that inform staff decision making and make up what Horlick-Jones (2005) describes as an ‘interactional matrix'. These include: local informal hierarchies, professional conflicts and local habits and rituals in the carrying out of caring tasks, all of which staff must negotiate in their daily working practices. The interplay of value systems that shape the actions and decision making of staff in their relationships with patients is complex and being sensitive to the context and contingencies of actions and decision making is essential to understanding how particular sets of values are able to take precedence.

In this case, once Nurse Peters attaches value to this man's needs, over the values of institutional processes, he uses the file in the reverse way to its proposed purpose. Nurse Peters decides upon the triage category that the patient was to be placed in and subsequently works backwards in the flow chart in order to present the correct signs and symptoms to legitimate this decision. As Berg (1992) similarly illustrated in his study of medical assessments, both patient histories and examination data can be given more or less validity depending on their usefulness in determining the desired transformation.

Both Nurse Claire and Nurse Peters illustrate how staff utilise the tools and techniques for clinical governance in unintended ways. The meaning attached to these systems and their interpretation of them in their interactions with patients maintains proximity and makes it possible for patients to be present as a moral demand.

## Discussion

This article highlights the practices of effacement that staff undertake to cope with the tensions they experience between responding to patients as full persons and responding to institutional concerns of accountability, resource rationalisation and the management of institutional risk. The article illustrates the various strategies through which the distancing of patients as a moral demand is accomplished. First, the perception of patients as a group to be managed is an important way in which patients become cognized (Bauman, 1990) and subsequently objectified as a group in opposition. Second, when individual patients attempt to make their claims distinct, as in the case of the young woman who was believed to have self-harmed, staff respond to a negatively constituted attribute, such as the patient being deemed responsible for the problem they attend with. These traits are constructed according to professional interests and institutional concerns and potentially reduce patients' personhood to a representation of this negative trait. The effacement of patients as full moral persons enables staff to act under competing pressures that also have the potential to deny or suspend their own status as moral beings.

It is the contention of this article that this space of demoralisation is accomplished through the organisation of social relations within the ED. The ED is a space of demoralisation because staff are left with few alternatives but to make sense of their experiences through the clinical, organisational and administrative systems of classification that mediate patient assessments. Practices of effacement provide staff with a tool enabling them to cope with working in a demoralised environment. Such practices therefore both respond to and further create a space of demoralisation. In recognising demoralisation as a social accomplishment, morality ceases to be understood as socially modular, distinct from everyday life. Instead, morality and moral actions are collaboratively produced; we reproduce or shift our moral understandings together, in many daily interactions of social life (Walker, 1998). The problem of proximity and staff's responses to it are therefore both responsive to and constitutive of the wider organisational cultures in which they are embedded.

The article has provided three examples from the ED in which patients remain present as a moral demand and in doing so highlights the mechanisms through which moral proximity is re-established. These examples are important to highlight that ED staff do not simply perform to institutional logics of efficiency and accountability. This work, along with others (for example, Bolton and Houlihan, 2009) attempts to move beyond the agency/control dualism in understanding the relationship between health-care workers and NPM systems. Instead, these examples illustrate the potential for resistance and the multidimensionality in the ways such ‘institutions of modernity' (Bauman, 1994) are experienced and perceived by medical staff (Brown, 2011). While recognising the multiplicity of staff interpretation, in all three examples, staff are informed by the systems of governance that mediate their work. Although staff act in contrast to the intended purposes of techniques of governance, they remain implicated in there continuation as significant determinants of social organisation. As a result, in order to accomplish proximity, staff must both accommodate these systems of governance while at the same time make decisions that contradict their purpose.

To return to the problem posed at the beginning of this article: What are the conditions in which a lack of care in institutions like the NHS is able to endure? The case of the ED provides some useful insight. The examples show that the ability of staff to draw on a morally grounded responsibility towards their patients is being challenged by the competing demands placed upon them. For staff to re-create moral proximity, their actions and interactions are necessarily in conflict with the ‘institutions of modernity' (Bauman, 1994) that govern their work, institutions that are increasingly embedded in health and social care organisations both in the United Kingdom and internationally (Schout *et al*, 2011). In other words, the value of ethically informed care is increasingly absent from the moral community of health-care work (Peter and Liaschenko, 2004). To sustain a moral response to those seeking help through the NHS, staff must actively resist the dominant cultures that shape their daily working practices. Furthermore, the enhancement of the moral and ethical orientation of caring work, that comes from challenging these cultures, rarely results in reward or social recognition.

Bauman and Fevre's theories of morality and demoralisation provide significant insight into the ‘problem of care' within the NHS. The value of utilising such concepts lies in their capacity to highlight the social and institutional challenges to proximity (Bauman, 1991, 1990) and to moral categories for sensemaking (Fevre, 2000) that form the pre-requisites for maintaining human social relationships that are responsive to the other. The entrenchment of institutional concerns into the daily practices of clinicians and health-care workers can be seen not just in the ED but across many areas of the NHS, where fears over increased rationing, financial and reputational risks and efficiency targets shape the daily lives of clinicians as well as managers (Maruthappu *et al*, 2010).

Developing these theories in the context of health care is important as it challenges individualistic explanations of the ‘problem of care' in the NHS by directing our attention towards the organisational and institutional cultures of health care and away from the ‘inner' morals of individual practitioners. Finally, theories of demoralisation are essential in enabling us to identify not just the distortions occurring in the relationships between health practitioners and patients (and the potential stripping away of patients' personhood), but also to highlight how these distortions deny or suspend staff's own status as moral beings.
